# Associative Effects between Forages and Concentrates on In Vitro Fermentation of Working Equine Diets

**DOI:** 10.3390/ani11082212

**Published:** 2021-07-26

**Authors:** Mónica Gandarillas, Juan Pablo Keim, Elisa María Gapp

**Affiliations:** 1 Facultad de Ciencias Agrarias y Alimentarias, Instituto de Producción Animal, Universidad Austral de Chile, Independencia 631, Valdivia 5110566, Chile; 2 Escuela de Graduados, Facultad de Ciencias Agrarias y Alimentarias, Universidad Austral de Chile, Independencia 631, Valdivia 5110566, Chile; elisa.gapp@alumnos.uach.cl

**Keywords:** equine, forages, concentrates and grain, enzymatic digestion

## Abstract

**Simple Summary:**

The aim of this paper is to evaluate post-gastric changes in the fermentability of mixtures of different forages and concentrated feeds that are representative of the traditional diets of high-performance horses using the in vitro gas production (GP) technique. Based on GP and volatile fatty acids (VFA), the post-gastric fermentation of concentrates is greater than that of forages. However, when we combined forages and concentrates, the GP parameters and VFA concentrations of some forage–concentrate mixtures had unexpected values in comparison to the fermentation of pure ingredients, indicating the occurrence of associative effects. This shows that there is a need to evaluate the fermentation of diets, rather than predicting from the values of pure ingredients.

**Abstract:**

Background: Horses are hindgut fermenters, and it is therefore important to determine the postgastric nutritive value of their feedstuffs and diets. Moreover, it has been demonstrated in other animal species that the fermentation of diets results in different values than those expected from pure ingredients. Therefore, the general objective of this work is to evaluate the gas production (GP) and volatile fatty acid (VFA) concentration, as well as the associative effects, of mixtures of different forages and concentrated foods, which are representative of the traditional diets of high-performance horses. Methods: An in vitro gas production experiment was conducted to assess the fermentation of two forages and three concentrates that are typical in horse diets. The combination of 70% of forage and 30% concentrates was also assessed to determine potential associative effects. Results: Concentrates and grains produced higher GP and VFA than forages when evaluated alone. When experimental diets were incubated, GP parameters and VFA concentrations of forage–concentrate mixtures had unexpected differences from the values expected from the fermentation of pure ingredients, suggesting the occurrence of associative effects. Conclusions: Our results indicate that there is a need to evaluate the fermentation of diets, rather than predicting from the values of pure ingredients.

## 1. Introduction

Horses are involved in diverse equestrian disciplines that increase their digestible energy and nutrient requirements; therefore, highly balanced nutrient and energy diets are required [1]. Thus, sport equine diets will need to be designed to ensure maintenance, work and, in some cases, growing requirements [2].

Sport horses are often housed for the majority of the day in stalls with limited access to pasture outdoors; instead, they are fed two or three meals per day containing forages and cereal grains or commercial concentrates [3]. In common equestrian disciplines, such as racing, polo, show jumping, dressage or eventing, their liveweight varies from 350 to 600 kg on average. Considering the NRC requirements for a 500 kg horse that works five to six days a week for 30 to 60 min of walking, trotting, cantering, and other special skills, a daily requirement of 34.5 Mcal of digestible energy (DE) was established [4]. Considering that an average horse will need to consume 2.25% to 2.5% of their bodyweight, it is expected that each kilogram of the diet must have a DE concentration of 2.76 to 3.0 Mcal/kg.

The most common diets in these situations consist of hay in addition to a source of concentrated digestible energy, such as cereal grains or a mixture of concentrate ingredients [3,5]. The most common hay forages fed to horses are grass, grass/legume, or alfalfa hay [3,6], which have 2.04, 2.25, and 2.45 Mcal DE/kg DM [4], respectively, which means that DE requirements of a hard working horse will not be met with hay only. For this reason, starch-rich feedstuffs are added to the diets of working horses. A series of surveys were given to owners of working horses and these showed that horse diets had around a 70% hay forage source and a 30% starch rich ingredient source [3,5,7]. Among hays, alfalfa hay, either in hay, cubes, or pellets, is the main source of forage, followed by grass hay. At the same time, oats, maize, barley, wheat, and wheat by-products (wheat middlings and wheat shorts), or a mix of them, with soybean and other micronutrients sources are used [2,3,5,8].

Horse feed has been evaluated using digestibility values measured in vivo, in vitro or were derived from a chemical analysis [9]. While the in vivo method is the most accurate, it is costly, time-consuming, labor-intensive, and needs to be performed directly on the animals [10]. In theory, this method should be ideal for measuring the nutrient digestibility in the animal; however, in vivo techniques require large amounts of feed and several experimental replicates in order to obtain reliable results. The cost of obtaining an adequate number of replicates is high, so when added to the costs of maintaining and sampling a large number of animals, in vivo studies may be difficult to access. In addition, in vivo research often requires surgically operated animals, thus compromising animal welfare to some degree. This has led to increased interest in the use of in vitro methods to estimate digestibility in the gastrointestinal tract [11].

In horses, various attempts have been used to develop an in vitro method to estimate digestibility. Bush et al. [12] used cecal liquid to evaluate the fermentation of feeds using the gas production (GP) technique. However, this method has as its weaknesses, including the need to use cannulated animals as cecal microflora donors. An alternative that replaces ruminal or cecal fluid and does not require surgical intervention is the use of equine feces, since they have fermentable activity that allows for their use as an inoculum for in vitro fermentation studies [13,14]. While the technique used by Theodorou et al. [14] initially relied on rumen fluid as a source of microbial inoculum, the use of feces has proven to be a successful alternative source of microbial inoculum in ruminant, equid, and other non-ruminant studies. The GP profiles are similar when inoculated with cecal liquor or feces, as shown in Reference [15]. Therefore, feces, as a microbial inoculum for in vitro digestibility studies in equines, has several practical and economic advantages over cecal fluid, since it does not require the use of surgically prepared animals and can be collected from any individual or from several animals, thus minimizing the influence of animal-to-animal variations [16,17].

The cumulative GP profiles of feeds have a wide diversity, demonstrating the potential of the pressure transducer technique to assess the digestion kinetics of feeds used in equines [18]. However, it is important to mention that in vitro techniques are not designed to accurately measure absolute digestibility, but rather to predict it and compare the relative digestibility of different feeds [14]. Gas production profiles and parameters are indicators of the rate and extent of the degradability of feedstuffs in the caecum and ventral colon of horses, while total VFA and the type of VFA are the fermentation end products from the microorganisms of materials that escape the enzymatic digestion and serve as energy sources for horses [19]. An increase in the propionate proportion may enhance the overall energy balance of the horse, given that propionate is glucogenic [20].

Based on the diets consisting of 70% hay and 30% concentrate, it can be deduced that diets comprising forage and concentrate/grain, which contain less ADF or NDF in their nutritional composition, will have a higher GP than diets that contain higher values of ADF and NDF. In contrast, it is expected that diets high in fiber will increase the concentration of acetic acid in relation to propionic and butyric acid. In contrast, high starch diets will increase propionic acid production. Studies usually evaluate pure ingredients, without additives and are inoculated with feces without prior enzymatic digestion [21,22]; however, there is limited information on the fermentation of horse diets. However, it has been observed that there are positive and negative associative effects when mixtures are incubated, as compared to the expected values from incubations of pure ingredients [23]. For example, it has been observed that including sugar beet pulp in horse diets has increased the degradability of the cell wall fraction of alfalfa, as compared to pure ingredients [24]. Thus, there is a need to assess the fermentation of mixed diets rather than pure ingredients.

Therefore, the general objective of this work is to evaluate the GP and volatile fatty acid concentrations that are assessed using the in vitro GP technique and the associative effects of mixtures of different forages and concentrated feeds, which are representative of the traditional diets of high-performance horses.

## 2. Materials and Methods

This study was carried out at the Animal Nutrition Laboratory of the Animal Production Institute and Institute of Food Science and Technology (ICYTAL) of Universidad Austral de Chile in February 2019. The protocol of this study was approved by the Bio-Ethics Committee of Universidad Austral de Chile 369/2019.

### 2.1. Treatments and Experimental Diets

Nine different treatments were designed. The nine treatments included the combination of 70% (on a dry matter (DM) basis) of three hay forages and 30% of three grains or concentrate sources, resulting in nine experimental diets. The selected forages and grains or concentrates were selected based on surveys previously published [3,5]. The sources of hay used were alfalfa hay (AH), a mix of alfalfa/grass hay (AGH, 50:50) and mature grass hay (GH). The concentrates or grains used were a mix of oats grain and wheat middlings (OWM, 50:50) and two commercial concentrates: a starch-based concentrate (CCA), composed of wheat bran, oats, soybean meal, corn, molasses, vegetable oil and salt, and a concentrate that included soluble fiber ingredients (CCB), which was composed of barley, oats, corn, wheat, triticale, rice, wheat middlings, dehydrated fruit and carrot, alfalfa, linseed, molasses, sunflower oil, and/or soybean meal. Due to the privacy of manufacturers of each ingredient included in the formulas, we decided to list the ingredients of CCA and CCB from the first to the last level of inclusion.

### 2.2. Ingredients and Chemical Composition

Chemical composition results of all ingredients are shown in Table 1. The dry matter content was measured by weighing the samples before and after they were dried with a forced-air oven, initially at 60 °C for 48 h and then at 105 °C for 12 h. Subsequently, the dried ingredients were mill ground through a 1-mm sieve and mixed homogeneously. Finally, each sample was stored in hermetic bags for further use. From each ingredient, 100 g were taken for chemical analysis. The crude protein concentration was determined by combustion (Leco Model FP-428, Nitrogen Determinator, Leco Corporation, St Joseph, MI, USA), based on the DUMAS method (nitrogen ×6.25) [25], neutral detergent fiber (aNDF) was measured using a heat stable amylase [26], and ash and ether extract (EE) were analyzed according to AOAC (Methods ID 942.05 and ID 920.39 for ash and EE, respectively) [27].

All the ingredients used for the experimental diets were treated with digestive enzymes using the in vitro method with the technique described by Strauch et al. [28]. Briefly, each sample was performed in triplicates. The first solution was prepared by mixing 400 mL of distilled water at 38 °C with a 1M HCl solution to adjust the pH to 3.2 ± 0.05. To homogenize each mixture, a constant magnetic stirrer was used where the temperature could also be controlled. Then, 20 g of the diets and the pure, dried, and ground ingredients were added to 2.28 g/L of Pepsin and were incubated in a water bath at 38 °C for 1 h under constant stirring. Subsequently, 4.4 g/L of monopotassium phosphate and 4.6 g/L of di sodium phosphate were added as a buffer. It was then homogenized and 1M sodium hydroxide was added to achieve a pH of 6.9 + 0.04. Finally, 0.25 g/L of pancreatin was added and further incubated in a water bath at 38 °C for 1 h under constant stirring. Then, the samples were filtered in a vacuum filter to separate the solid residue of the digestion and then frozen and lyophilized to determine the enzymatic digestibility.

### 2.3. In Vitro Incubations

The procedure described by Theodorou et al. [15] was followed. Duplicates of the 5 ingredients (10 bottles) and triplicates of the 9 experimental diets (27 bottles), plus two blanks, were made (39 bottles in total for each incubation run). Each triplicate from the enzymatic digestibility samples was incubated in a different run and completed three incubation runs. Pure ingredients and mixture samples (1 g) were added into 160 mL glass bottles. Subsequently, 85 mL of Goering-Van Soest medium and 4 mL of the reducing agent (NaOH 2.5 mM and cysteine-HCl 2.5 mM) were added at 39 °C under continuous gasification (CO_2_) in order to maintain anaerobic conditions, and the bottles were covered with rubber stoppers and aluminum seals.

Approximately 2 kg of feces were collected directly from the rectum of three 600 kg BW show jumping geldings through rectal palpation in order to avoid contamination from the soil. The horses were housed at an equestrian club, located 10 km away from the Animal Nutrition laboratory and were fed with the same diet (12 kg DM of 35% of AH, 35% GH, 10% CCA, 10% CCB, and 10% O-WM). The feces were transported in thermal flasks, which were filled with hot water (60 °C) before being used, and were emptied before the feces were added in order to maintain the temperature of the fecal microbes. The feces of the horses were pooled and subsequently filtered through a double-layer muslin strainer. The liquid from the filtration was recovered in a glass beaker with a constant flow of CO_2_ to maintain anaerobic conditions.

Then, fecal fluid was inoculated (10 mL) into the bottles. After inoculation, the bottles were placed in a water bath at 39 °C under continuous horizontal movement at 50 rpm.

Once the fecal fluid was inoculated, the initial gas was extracted from the bottles. The gas pressure in the headspace of the bottles, above atmospheric pressure, was measured manually with a pressure transducer (PCE Instruments, Tobarra, Albacete, Spain) at 2, 4, 6, 8, 12, 18, 24, 36, 48, 60, 72, and 96 h. The volume of the gas produced was measured by extraction using syringes connected by a 3-way Luer valve from the bottles until the visual display of the transducer read zero, and once the volume of gas produced was recorded, it was withdrawn. Fermentations were stopped after 96 h by placing the bottles on ice. A total of three incubation runs were carried out, each corresponding to one triplicate from enzymatic digestions (block factor).

Once the in vitro incubation was finished, the samples were kept on ice to stop the fermentative processes. Residue duplicates and triplicates from experimental diets and pure ingredients were pooled and centrifuged at 15,000× *g* and 4 °C. After centrifugation, 0.9 mL of the supernatant was extracted in order to determine the VFA concentrations with a GG-2010 gas chromatograph (Shimadzu Corporation, Kyoto, KYT, Japan).

### 2.4. In Vitro GP Kinetics

After correcting the GP of the blanks, the obtained GP data were adjusted to the generalized Michaelis–Menten model without a lag phase [29] as follows:

GP = A × [*T^n^*/(*T^n^* + *K^n^*)] (1)

where GP is the GP at time *T*; A is the asymptote of GP (mL); *n* is the determined value of the shape of the curve; and *K* is the time to produce half of A.

The following parameters were calculated according to Groot et al. [30] and France et al. [29], and are as follows:

fermentation rate at half-life (C) = *n*/(2 × *K*) (2)

maximal fermentation rate (MDR) = (*n* − 1)^(*n* − 1)/*n*)/*k*^ (3)

### 2.5. Experimental Design and Statistical Analysis

Analytical replicates of GP data were averaged and then fitted to the generalized Michaelis–Menten model without a lag phase using the NLIN procedure of SAS [31] in order to determine the GP, A, K, and *n* parameters. Data were analyzed using a randomized complete block design with a 3 × 3 factorial arrangement of treatments, with forage and grain-concentrates as the main factors and the random effect of the incubation run as a block; we used the MIXED procedure of SAS [31]. When significant differences (*p* < 0.05) were found, the Tukey–Kramer multiple-comparison test was used in the LSMEANS procedure statement in SAS. To evaluate the associative effects of substrates on fermentation parameters, the percentage differences between the values measured for the forage/concentrate combinations and the calculated balanced values was calculated as follows: % difference = 100 × [(observed value − calculated value)/calculated value], where the calculated value was the result of the relative proportion (70% forage and 30% grain-concentrate) of the observed values of the pure ingredients. Positive or negative values indicated positive or negative associative effects between ingredients in the mixture, respectively [32].

## 3. Results

### 3.1. In Vitro GP and Volatile Fatty Acids from Fermentation of Pure Ingredients

**Figure 1:**
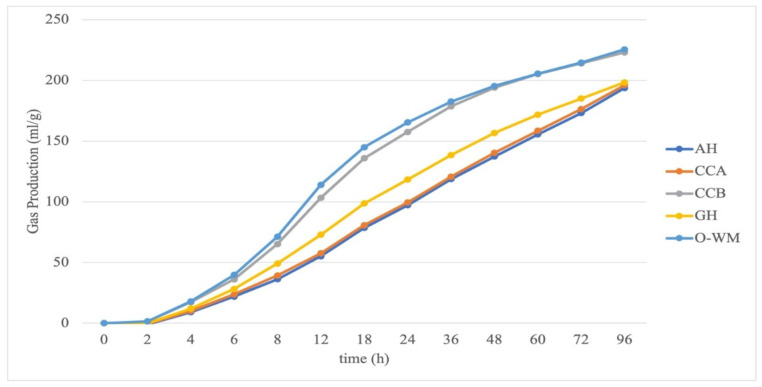
In vitro gas production kinetics of ingredients. AH: alfalfa hay, CCA: starch-based commercial concentrate, CCB: commercial concentrate with soluble fiber sources, GH: grass hay; O-WM: 50% oats and 50% wheat middlings.

Gas production kinetics of the pure ingredients are presented in Figure 1. The ingredients that obtained the greatest GP (O-WM and CCB) produced more gas consistently from 2 h of incubation to 96 h, whereas AH, GH, and CCA produced less gas throughout the incubations.

Asymptotic GP production did not differ between forages and concentrates or within forages (*p* > 0.05), whereas it was greater for CCA within concentrates, as compared to CCB and O-WM (Table 2). Concentrates (214.9 mL/g DM) produced more gas than forages (196.0 mL/g DM) after 96 h of incubation; no differences were observed between forages, and CCB and O-WM produced more gas than CCA. The time required to produce half of A did not differ between concentrates and forages or within forages, whereas for concentrates of CCA, it was reached 20 h later than CCB and O-WM. Parameters of the gas production rate (c and MDR) were both different when comparing forages and concentrates, within concentrates and within forages. Forages ferment slower than concentrates, AH was faster than GH, and CCB and O-WM were faster than CCA. The in vitro end pH was greater for forages than for concentrates (6.53 and 6.42; *p* < 0.001), and greater for GH, as compared to AH and GH-AH, whereas among the concentrates, CCA and CCB had a lower pH than O-WM. In vitro concentrations of VFA, molar percentages of acetate (C2), propionate (C3), and butyrate (C4) did not differ between forages and concentrates, within forages or within concentrates (*p* > 0.05). Nevertheless, fermentation of forages resulted in a greater acetate to propionate ratio (C2/C3), whereas no differences were observed within forages or within concentrates (*p* > 0.05).

### 3.2. In Vitro GP Kinetics and Volatile Fatty Acids of Fermentation from the Experimental Diets

**Figure 2:**
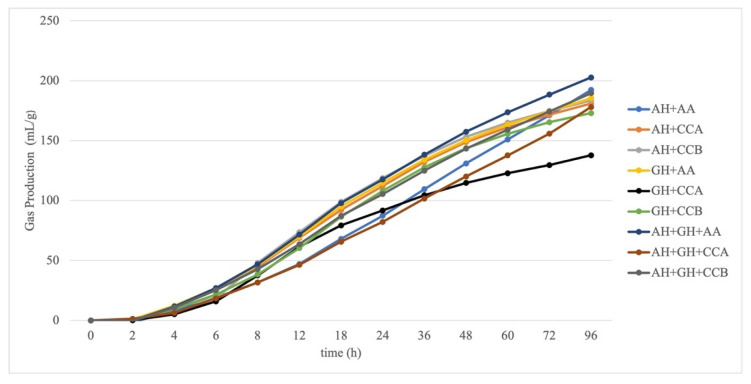
In vitro gas production kinetics of the experimental diets. AH: alfalfa hay, CCA: starch-based commercial concentrate, CCB: commercial concentrate with soluble fiber sources, GH: grass hay; O-WM: 50% oats and 50% wheat middlings.

The measured GP of the experimental diets is presented in Figure 2. The total GP was higher in the forage mixture (AH and GH) that was combined with O-WM and CCB from 2 h of incubation until 96 h (*p* < 0.05).

In vitro GP parameters (A, 96GP, k, c and MDR) were affected by a forage/concentrate interaction (*p* < 0.001). When mixed with AH, CCA and CCB showed a similar A and was lower than O-WM, whereas CCA had a lower A compared to CCB and O-WM when mixed with GH; finally, CCA had a greater A than CCB and O-WM when mixed with AH and GH. For 96GP, there were no differences between concentrates when mixed with AH and AH-GH, whereas a greater GP was observed for CCB and O-WM when compared to CCA. Fermentation rate parameters (c and MDR) were greater for CCA and CCB when mixed with AH and GH; however, when mixed with AH-GH, it was reduced for CCA and was greater for CCB and O-WM. This resulted in a longer k for O-WM when mixed with AH, and this was similar among the different types of concentrates with GH and was longer for CCA with AH-GH (Table 3).

In vitro pH and VFA were not affected by the type of forage, concentrate, or the interaction between them (*p* > 0.05). There were strong forage/concentrate interactions for acetate, propionate, butyrate percentages of VFA, and the acetate/propionate ratio. Acetate was similar between concentrates when combined with AH, but was greater for CCB when combined with GH and for O-WM when combined with AH-GH. Conversely, propionate was greater for CCA when mixed with AH, CCA, and CCB with AH-GH, and there were no differences among the concentrates when combined with GH. Butyrate was similar between concentrates when mixed with AH, greater for CCA, followed by O-WM, and lower for CCB with GH and greater for CCA and CCB than O-WM when mixed with AH-GH. The acetate/propionate ratio was similar between concentrate types when mixed with AH and GH, whereas it was greater for CCB and O-WM than CCA when combined with AH-GH.

Compared to the expected values of the pure ingredients, 96GP was reduced for CCB and O-WM, whereas fermentation rates increased for CCA and CCB by 50% and 35%, respectively, but reduced for O-WM by more than 100% when mixed with AH. When combined with GH, the 96GP was reduced by 40% for CCA, and 19% and 11% for CCB and O-WM, respectively; the fermentation rate increased for CCA, was unaffected for CCB and was reduced for O-WM. Gas production of CCA, mixed with AH-GH, was reduced by 10%, as compared to the expected values, whereas for CCB and O-WM, AH-GH was similar to the expected values; the fermentation rate reduced by 116%, 57%, and 34% for CCA, CCB, and O-WM, respectively.

The final pH values of the diets were close to the expected values from the calculations based on pure ingredients. When mixed with AH and GH, all the types of concentrates showed a slight positive effect, which ranged between 0% and 6% for the VFA concentration, whereas CCA and CCB showed a negative effect when combined with AH-GH and O-WM increased by 11%. The observed acetate concentration increased from 3% to 7% in all the dietary treatments, while the propionate concentration was 3% to 10% lower than the expected values. The observed concentration of butyrate was reduced by ca. 10%, except for CCA with GH and CCB with AH-GH, which showed reductions of 4% and 3%, respectively. The acetate/propionate ratio was 7% to 15% greater than the expected values.

## 4. Discussion

This is one of the series of experiments that used Theodorou’s in vitro technique to evaluate the nutritional value of some common feedstuffs fed to equine [22,23,33]. Our results showed that GP parameters and the VFA concentrations of some for-age-concentrate mixtures had different than expected values from the fermentation of pure ingredients, suggesting the occurrence of associative effects. This indicates that there is a need to evaluate the fermentation of diets, rather than predicting from the values of pure ingredients.

### In Vitro Fermentation

According to Mauricio et al. [34] and Lowman et al. [33], the accumulated gas profiles are similar when inoculated with cecal liquid or feces. Something similar happens in ruminants, with the difference being that there is a delay time in the GP when feces are used, as compared to ruminal liquor.

The horse is a hindgut fermenter, with enzymatic digestion of nutrients occurring before the fermentation process, which is carried out in the colon and cecum. For this reason, to obtain more accurate results in this study, a previous enzymatic digestion of the experimental diets was carried out. Abdouli and Attia compared the fermentation of the same horse feed with and without prior enzymatic digestion and found that the GP of the enzymatically pre-digested feeds tended to decrease as the feeds had more substrate that was sensitive to pepsin and amylase, while the GP of the feeds without pre-digestion was higher [17]. These results showed that the GP from non-predigested feeds overestimates their fermentation potential, and that when evaluating them, their fermentation capacity is overestimated, since the volume of the gas produced reflects the fermentation potential of the fiber fraction. Crude protein, as a result of the addition of pepsin, can be highly degraded under in vitro conditions [35,36]. The decrease in sugars, such as glucose, fructose and sucrose, which are easily digested and absorbed in the small intestine, unlike fiber, which is not degraded by endogenous enzymes, was also demonstrated [37]. These observations also confirm the importance of prececal digestive processes in horses when simulating in vitro digestion [26].

The greater GP and VFA concentrations, as well as the faster fermentation rate of concentrates than forages, suggests that the undigested fraction of concentrates is more fermentable than that of forages. These results are in accordance with References [22,38], which reported greater GP and fermentation rates in concentrates. In addition to our study, the same authors also observed differences among the different types of forages and types of concentrates. It has also been demonstrated that feeds incubated with fecal inoculums collected from ponies fed a high concentrate diet were degraded to a greater extent than samples incubated in fecal inoculums from those fed with high fiber diets [16,39]. These differences in the degradation rate may be attributable to the chemical composition of these ingredients, since, in general, forages contain more NDF that ferments more slowly than starch [40,41,42]. These findings further support the existing literature on the rapid degradation of nonstructural carbohydrates (NSC) in the horse large intestine environment and the slower degradation of feeds containing structural cell wall material [43]. Earing et al. [16] showed that NDF degradation was greater at 60 h than at 45 h; therefore, to ensure complete NDF degradation, a longer incubation period is necessary.

As opposed to that found by Fehlberg et al. [44], we observed no differences in the relative percentages of VFA, which may be explained by methodological differences among experiments. Those authors did not perform any enzymatic digestion prior to in vitro incubation, which has shown to modify the results from in vitro incubation in horse diets [17].

Through fermentation, the fibrous components are degraded to monosaccharides. The anaerobic environment of the hindgut prevents the complete oxidation of monosaccharides and instead produces VFA, acetate, propionate, and butyrate [45]. The results from previous studies indicated that changing the energy source from starch-rich feeds to high-fiber feeds in training horses changes the muscle energy supply from glucose to acetic acid due to increased fermentation in the hindgut [46] and the acetate/propionate ratio increases when the forage/concentrate ratio increases [47]. The experimental diet produced greater acetate concentrations, as compared to propionate. These results are supported by a study that demonstrated that hay (timothy and meadow fescue mix) positively influences the plasma acetate concentration [46,48]. Mixtures, including CCA, resulted in greater propionate concentrations. It has been observed that the high levels of starch that enter the posterior intestine may cause harmful changes in the gastrointestinal tract [49], since they produce a rapid fermentation and therefore a rapid production of VFAs. The rapid production of VFAs has been reported to overload the pH control mechanisms exerted by buffer secretion and acid absorption [50], and consequently, the pH of the hindgut decreases, resulting in the rapid growth of lactate-producing gram positive bacteria [31,36], causing microbial disorders that can lead to clinical disorders, such as laminitis and colic [51,52].

Diets that are high in crude protein (14% to 17%) have been shown to increase the buffering capacity of the rumen [53], indicating that the protein in food may act as a buffer against acidity. Proteins have side chains that can gain or lose protons, and thus serve as pH buffers and act against the drop in hindgut pH as a result of a starch overload [54]. Alfalfa is a legume known to have a high buffering capacity due to its high levels of organic acids and proteins [55]. In addition, alfalfa has been reported to reduce the damaging effects of high NSC diets on gastric pH, which is due to the buffering capacity of proteins that neutralizes the capacity of short chain fatty acids to reduce pH and produce ulcerations [36]. In this experiment, it was observed that the diets containing alfalfa had a higher pH value than the other mixtures, which may be due to the buffering capacity mentioned above. This high buffering capacity of alfalfa can be used to counteract the acidotic effects associated with a high intake of NSC in the horse’s large intestine. However, there is currently no information to support this statement [35]. For this reason, it is important to achieve a good balance in the diets of horses in general, since any type of imbalance can produce harmful changes in their gastrointestinal system.

In addition, it is important to mention that other studies found that the inclusion of soluble fiber, such as feeds rich in pectins, resulted in an increase in cecal VFA and a propionate concentration, which consequently resulted in stable blood glucose and insulin responses, and these were constant. The results suggested that fiber-based diets high in soluble fiber could meet the energy requirements of horses at the medium working level [47]. This is possible since the soluble fiber fraction is fermented to propionic acid through the succinate pathway without the formation of lactic acid, as in the case of high starch diets [47]. In our experiment, CCB contained soluble fiber sources, such as dehydrated fruits and carrots, but tended to reduce propionate, as compared to CCA (starch-based concentrate), except when combined with AH-GH. This suggested that the forage source influences the fermentation of concentrates.

Regarding the experimental diets, there was a variation between GP kinetics and the chemical composition of them. In this trial, the GP was higher in diets comprising forage (mix AH-GH, AH, GH) mixed with O-WM and CCB (Figure 2). In the case of the experimental diets that contain O-WM, the higher GP could be explained by the greater amount of NDF and ADF that they contain, as well as CCB, since the latest was composed of several grains, plus dehydrated apple and carrot in addition to alfalfa hay in pellets, which were not enzymatically digested in the preincubation, whereas residues of CCA may be less fermentable. Thompson et al. [56] showed that there are associated effects in several nutrients, so it does not depend on the sum of the individual components. Murray et al. [21] observed that including sugar beet pulp in horse diets increased the degradability of the cell wall fraction of alfalfa, as compared to the pure ingredients.

While there were some similarities in the total GP between the different experimental diets inoculated in this study, the GP rate varied considerably and appeared to be substrate-dependent [57]. When different substrates are incubated in the same container, it is possible that the digestion of one substrate may influence the digestion of another substrate by altering the microbial populations present in the inoculum [58,59,60]. This was reflected in our results, where the GP parameters and VFA concentrations of some forage–concentrate mixtures produced different than expected values from the fermentation of pure ingredients. Propionate and butyrate concentrations were lower when mixed. Moreover, when concentrates were combined with GH, the values were reduced to a larger extent, as compared to AH and AH-GH. These associative effects have been reported previously for ruminants [32], but to the best of our knowledge, have not been reported for horses and needs to be considered when formulating horse diets.

## 5. Conclusions

It was possible to evaluate the in vitro GP parameters of the five common ingredients fed to equine, and nine experimental diets with 3 × 3 combinations of 70% forage and 30% concentrate that was inoculated with horse feces after being enzymatically digested. While we found a variation in the fermentation parameters among ingredients and among different experimental diets inoculated in this study, concentrates and grains produced higher GP and VFA than forages when evaluated alone. In the experimental diets, the GP at 96 h of incubation was higher in the mixtures of the different forages that included oats-bran. When comparing the pH values, the mixtures containing alfalfa obtained higher values. Finally, our results showed that GP parameters and VFA concentrations of some forage–concentrate mixtures produced different than expected values from the fermentation of pure ingredients, suggesting the occurrence of associative effects. This indicates that there is a need to evaluate the fermentation of diets, rather than predicting from values of pure ingredients.

## Figures and Tables

**Table 1:** Chemical composition and enzymatic digestibility of ingredients used for experimental diets (g/100 g DM).

Ingredient	Dry Matter	Crude Protein	NDF	ADF	Ether Extract	Ash	Ezymatic Digestibility
Grass hay	87.50	11.86	53.66	34.50	0.91	3.43	33.90
Alfalfa hay	88.50	17.65	45.81	37.02	1.32	4.32	31.20
Comercial concentrate A	88.50	14.61	24.61	11.21	3.82	5.35	55.50
Comercial concentrate B	86.50	11.31	23.88	10.73	2.07	2.69	58.90
Oats/wheat middlings	87.50	12.83	35.66	13.11	5.41	2.87	54.80

**Table 2:** In vitro GP parameters and concentrations of volatile fatty acids produced in the fermentation of the pure ingredients used for the experimental diets.

	Forages		Concentrate				*p* Value		
	AH	GH	AH-GH	CCA	CCB	O-WM	Concentrates v/s Forages	Within Forages	Within Concentrates
A	220.2	252.9	236.6	257.9	224.7	220.6	0.838	0.061	0.007
GP96	198.3	193.7	196.0	196.1	223.0	225.5	0.023	0.641	0.005
K	22.3	39.0	30.7	38.5	14.3	12.8	0.204	0.068	0.001
C	0.031	0.016	0.0235	0.017	0.06	0.07	0.046	0.0395	<0.001
MDR	0.035	0.021	0.028	0.02	0.06	0.07	0.047	0.0291	<0.001
pH	6.52	6.55	6.54	6.40	6.38	6.47	<0.001	0.008	0.009
VFA	67.6	69.7	68.7	104.7	109.5	94.2	<0.001	0.4271	0.538
C2	58.8	59.7	59.3	55.1	57.3	57.9	0.258	0.475	0.194
C3	23.2	23.1	23.2	26.4	26.7	23.2	0.649	0.931	0.338
C4	18	17.2	17.6	18.4	16.2	18.4	0.423	0.39	0.194
C2/C3	2.54	2.58	2.56	2.08	2.14	2.43	0.022	0.805	0.339

AH: alfalfa hay, AH-GH: 50% alfalfa hay and 50% grass hay, CCA: starch-based commercial concentrate, CCB: commercial concentrate with soluble fiber sources, GH: grass hay; O-WM: 50% oats and 50% wheat middlings; A: asymptotic gas volume (mL/g MS); K: time to produce half of A (h); C: degradation rate at half the asymptote (/h); MDR: maximum degradation rate (/h); GP6: gas production at 96 h; VFA: volatile fatty acids (mmol/L); C2: acetate (mol/100 mol); C3: propionate (mol/100 mol); C4: butyrate (mol/100 mol).

**Table 3:** Forage concentrate interaction and associative effects for in vitro gas production parameters and concentration of volatile fatty acids produced.

	Alfalfa Hay						Grass Hay						50% Alfalfa Hay–50% Grass Hay						
	CCA		CCB		O-WM		CCA		CCB		O-WM		CCA		CCB		O-WM		*p* Value
A	196.8	−29%	190.9	−28%	283.9	14%	136.5	−70%	184.9	−20%	200.2	−10%	268.9	10%	223.6	−4%	228.6	−1%	<0.001
GP96	192.3	−1%	183.6	−10%	192.3	−6%	137.8	−43%	173	−19%	185.33	−11%	178	−10%	189.6	−8%	202.7	−1%	<0.001
K	20.6	−88%	18.3	−73%	51.8	40%	15.6	−74%	20.35	2%	20.43	5%	55	40%	27.6	7%	24.1	−5%	<0.001
C	0.037	56%	0.047	38%	0.011	−193%	0.055	51%	0.04	1%	0.036	−19%	0.01	−116%	0.022	−57%	0.028	−34%	<0.001
MDR	0.04	48%	0.048	32%	0.015	−138%	0.057	46%	0.042	−1%	0.039	−17%	0.015	−71%	0.028	−34%	0.032	−27%	<0.001
pH	6.48	0%	6.49	0%	6.49	−1%	6.43	−1%	6.43	−1%	6.41	−1%	6.45	−1%	6.44	−1%	6.46	−1%	0.099
VFA	81.9	2%	87.3	6%	77.3	0%	81.5	3%	85.6	6%	83.1	9%	77.1	−3%	77.3	−5%	85.4	11%	0.058
C2	60.7	4%	62.1	5%	61.3	3%	60.1	4%	62.3	6%	61.6	5%	60	3%	60	2%	63	7%	0.003
C3	23.4	−3%	22.4	−8%	22.7	−3%	22.5	−7%	22	−10%	21.9	−7%	23.4	−3%	23.4	−3%	21.7	−8%	0.021
C4	15.8	−11%	15.5	−9%	16	−10%	17.4	−4%	15.6	−12%	16.5	−10%	16.6	−7%	16.7	−3%	15.4	−16%	0.001
C2/C3	2.6	7%	2.78	12%	2.7	6%	2.68	10%	2.84	15%	2.81	11%	2.56	6%	2.56	5%	2.9	13%	0.011

CCA: starch-based commercial concentrate, CCB: commercial concentrate with soluble fiber sources; O-WM: 50% oats and 50% wheat middlings; A: asymptotic gas volume (mL g^−1^ MS); K: time to produce half of A (h^−1^); C: degradation rate at half the asymptote; MDR: maximum degradation rate; PG96: gas production at 96 h; VFA: volatile fatty acids (mmol L^−1^); C2: acetic (mol 100 mol^−1^); C3: propionic (mol 100 mol^−1^); C4: butyric (mol 100 mol^−1^).

## Data Availability

The data presented in this study are available on request from the corresponding author.
